# Oral mucosal lesions and their association with tobacco use and qat chewing among Yemeni dental patients

**DOI:** 10.4317/jced.51706

**Published:** 2014-12-01

**Authors:** Sadeq A. Al-Maweri, Nader A. Alaizari, Ghadah A. Al-Sufyani

**Affiliations:** 1 Assistant professor, Department of Oral Medicine, Faculty of Dentistry, Sana’a University, Yemen; 2 Lecturer, Department of Oral Medicine, Faculty of Dentistry, Sana’a University, Yemen; 3 Dental practitioner, Department of oral and dental surgery, Al-Kuwait teaching Hospital, Sana’a, Yemen

## Abstract

Objectives: This study aimed to assess the prevalence of oral mucosal lesions (OMLs) in patients attending outpatient dental clinics in Sana`a university, Yemen, and to evaluate the association of such lesions with age, gender, and oral habits. Materials and Methods: This cross-sectional study included 409 subjects (272 males, 137 females, age: 15-86 years). Detailed clinical examination was performed in accordance with international criteria. Patient history included age, gender, education, denture wearing and risk habits (tobacco use and qat chewing). Data were analyzed using SPSS 19.00. Results: The prevalence of OMLs was 58.4% with a significant difference between men (63.6%) and women (48.2%; P < 0.05). The most common lesions were fissured tongue (37.2%), hairy tongue (15.9%), tumors and tumor-like lesions (9.8%), qat-induced white lesions (9.3%) and racial pigmentation (5.9%). Overall OMLs prevalence was linked to risk habits and age; qat chewing was statistically significant risk factor for having fissured tongue (OR: 1.77), hairy tongue (OR: 2.74), and white lesions (OR: 2.39) (P < 0.05). Cigarette smoking was statistically significant risk factor for having hairy tongue (OR: 2.82), white lesions (OR: 3.60) and tumors and tumor-like lesions (OR: 2.91) (P < 0.01). The increase in age was statistically significant risk factor for having tumors and tumor-like lesions (OR: 1.04; P < 0.001). Conclusions: The current results indicate that the occurrence of OMLs among Yemeni adults is high and emphasize that risk habits and age have some relationship with the presence of OMLs.

** Key words:**Oral mucosa, oral lesions, prevalence, smoking, qat chewing.

## Introduction

Epidemiological studies can provide an important vision for understanding the prevalence, extent and severity of oral diseases in the population (1,2).

The prevalence of OMLs varies by geographic location and has been reported 9.7% in Malaysia (3), 15.5% in Turkey (2), 61.6% in Solvania (4), 15.0% in Saudi Arabia (5), 19.4% in Iran (6), and 58.1% in Kuwait (7). The data obtained from these oral health surveys are essential for the establishment of adequate preventive and healthy promotion measures.

In addition to smoking, qat or khat chewing (a widespread social habit in Yemen) and smokeless tobacco (locally known as shammah) are highly prevalent habits in Yemen, (8). Both habits have been linked with occurrence of benign and malignant mucosal lesions (8-10).

Unfortunately, in Yemen, only very few studies have been conducted on oral mucosal lesions, and most of these studies were mostly about the association between white lesions and qat chewing (10,11); those studies did not include all oral lesions in adult populations. The pattern of the disease is also changing due to increasing awareness, changes in lifestyle and increasing interest in oral health. Moreover, there is a great need for clinical studies to establish baseline data on the prevalence of oral lesions. Therefore, this study was carried out to assess the prevalence and association of oral mucosal lesions with gender, age, and oral risk habits among Yemeni adults visiting the dental school of Sana’a University.

## Patient and Methods

The present study was approved by the Research and Ethics Committee of the Faculty of Medicine and Health Sciences, Sana’a University, Yemen. All volunteers were informed about the aims and methods of the study, and written consents were obtained.

This cross-sectional study was performed over 3 months period from May to October 2013 on 409 dental patients who were attended in the outpatient dental clinics at the College of Dentistry, Sana’a University, for an oral examination and treatment plan. The study sample included subjects who were 15 years and older. The sample size of the study was calculated using a single proportion formula, which was based on the proportion of oral lesions in Saudi dental patients from a previous study (5). The precision was set at 0.05 with a 95% confidence interval and a Z score of 1.96.

Visual examination of the mouth was carried out by an oral medicine specialist having more than 6 years of experience in diagnosis of oral mucosal lesions. Extra- and intraoral examination was performed under electrical overhead lights using a mouth mirror, tweezers, gauze, and a wooden tongue depressor. Any abnormality of the oral mucosa was diagnosed according to the diagnostic criteria described in the WHO guide to epidemiology and diagnosis of oral mucosal diseases (12). Recurrent herpetic lesions and aphthous stomatitis were recorded only if observed at the time of the examination. No biopsies or laboratory tests were done in the present study. The number of natural teeth and presence of dentures, either partial or complete, were also recorded. After the oral examination, patients who presented with lesions were referred for appropriate treatment.

Prior to clinical examination, demographic characteristics and clinical information including age, sex, oral risk habits, oral hygiene practices, systemic health, and history and current use of medications were recorded for all subjects.

SPSS (SPSS Inc., IL, Chicago, USA) version 19.00 was used for data entry and analysis. The Pearson chi-square test and Fisher’s exact test were used to assess differences in the incidence of oral mucosal lesions or conditions in relation to age, and sex.

Additionally, multiple logistic regression was used to assess the association of the oral lesions with age, gender, smoking and qat chewing; preliminary analysis was carried out using a univariable model, and variables showing associations with *P* < 0.25 were selected for the multivariable analysis. *P*-values < 0.05 were considered statistically significant.

## Results

Of the 409 subjects participated in the study, 272 (66.5%) were men and 137 (33.5%) were women. The mean age of the subjects was 39.26 ± 18.53 years (range 15-86 years); the majority of the subjects (55.7%) were in the 15-39 year age group. With regard to risk habits, 57.5% were qat chewers, 23.7% cigarette smokers and 6.6% smokeless tobacco users ( Table 1).

**Table 1:**
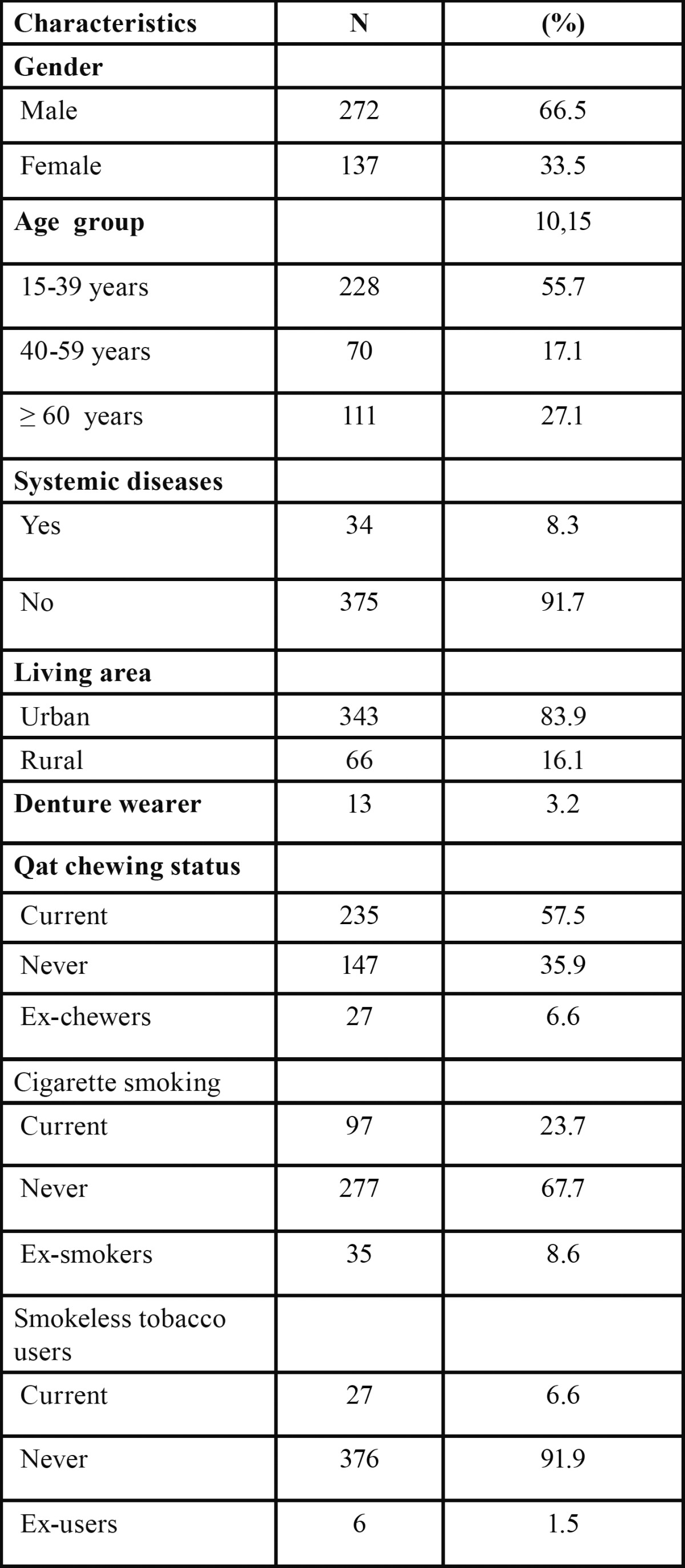
Demographic and characteristics of the study subjects.

Two hundred thirty nine of the subjects (58.4%) were diagnosed with at least one oral mucosal lesion at the time of examination. The prevalence rate was significantly higher in men than in women (63.6% vs 48.2%; *P* < 0.01). Twenty four types of OMLs observed were categorized into seven main groups: tongue lesions, white lesions, denture-related lesions, ulcerative lesions, exophytic lesions, pigmentation lesions, and miscellaneous. The most prevalent lesions were fissured tongue (37.2%), hairy tongue (15.9%), benign tumors (9.8%) and qat-induced white lesions (9.3%) ( Table 2).

**Table 2:**
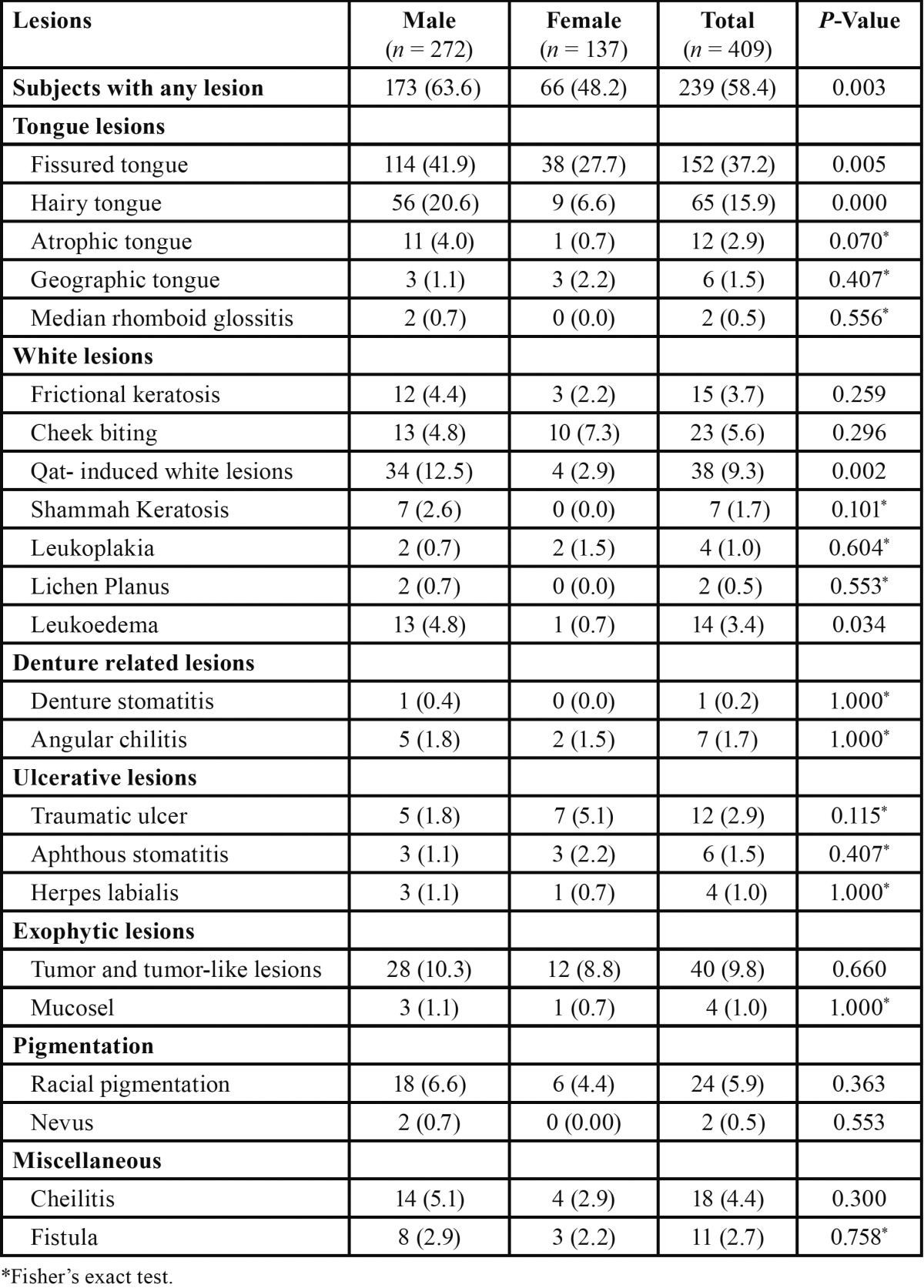
The frequency of oral lesions according to gender N (%).

There were some differences in the distribution of oral mucosal conditions among sexes. Fissured tongue, hairy tongue, qat-induced white lesions and leukoedema were significantly associated with men (*P* < 0.01, *P* < 0.001, *P* < 0.01, and *P* < 0.05, respectively). Atrophic tongue showed a weak association with sexes (4.0% in men and 0.7% in women; *P* = 0.07). Cheek biting and geographic tongue were found to be more common among women; however, these results were not statistically significant (*P* > 0.05) ( Table 2).

 Table 3 shows the distribution of various OMLs in relation to age. There was a significant increase in the incidence of, fissured tongue, leukoplakia, and exophytic lesions with age. The frequency of benign tumors was significantly higher in the 60 years and older group (*P* < 0.001), whereas fissured tongue and leukoplakia were more frequent in the 40-59 years group (*P* < 0.01 and < 0.001, respectively). On the other hand, cheilitis was significantly associated with younger age group (*P* < 0.001).

**Table 3:**
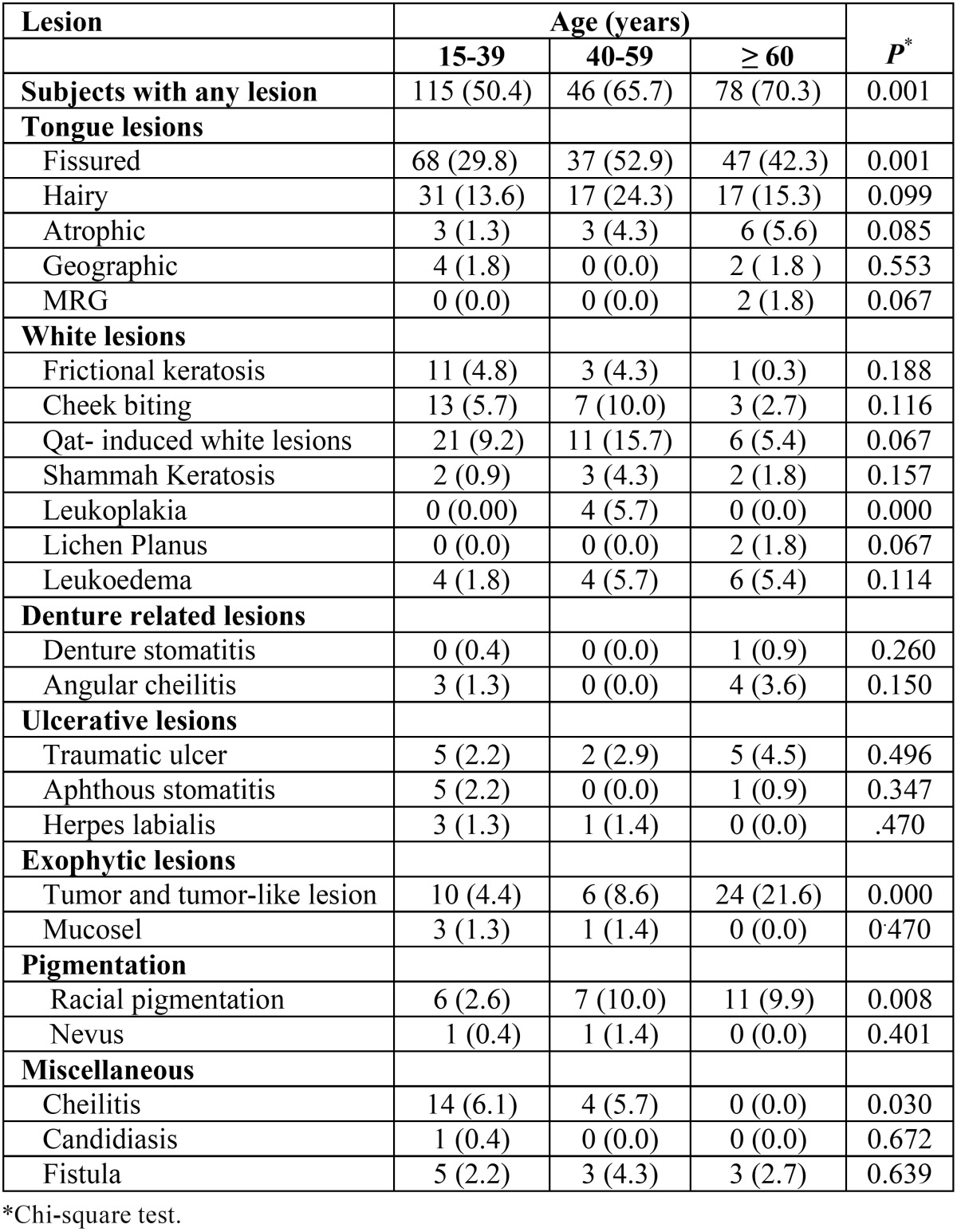
The frequency of oral lesions by age group N (%).

Logistic regression model was constructed to analyze the variables associated with more probability of having fissured tongue, hairy tongue, white lesions and tumors and tumor-like lesions. The independent variables included in the multivariable analysis were: age, gender, smoking and qat chewing. The results of logistic regression analysis presented in  Table 4. As seen in the table, qat chewing was statistically significant risk factor for fissured tongue (Odds ratio [OR]: 1.77), hairy tongue (OR: 2.74), and white lesions (OR: 2.39) (*P* < 0.05). Cigarette smoking was a statistically significant risk factor for hairy tongue (OR: 2.82), white lesions (OR: 3.60) and tumors and tumor-like lesions (OR: 2.91) (*P* < 0.01). The increase in age was a statistically significant risk factor for having tumors and tumor-like lesions (OR: 1.04, *P* < 0.001).

**Table 4:**
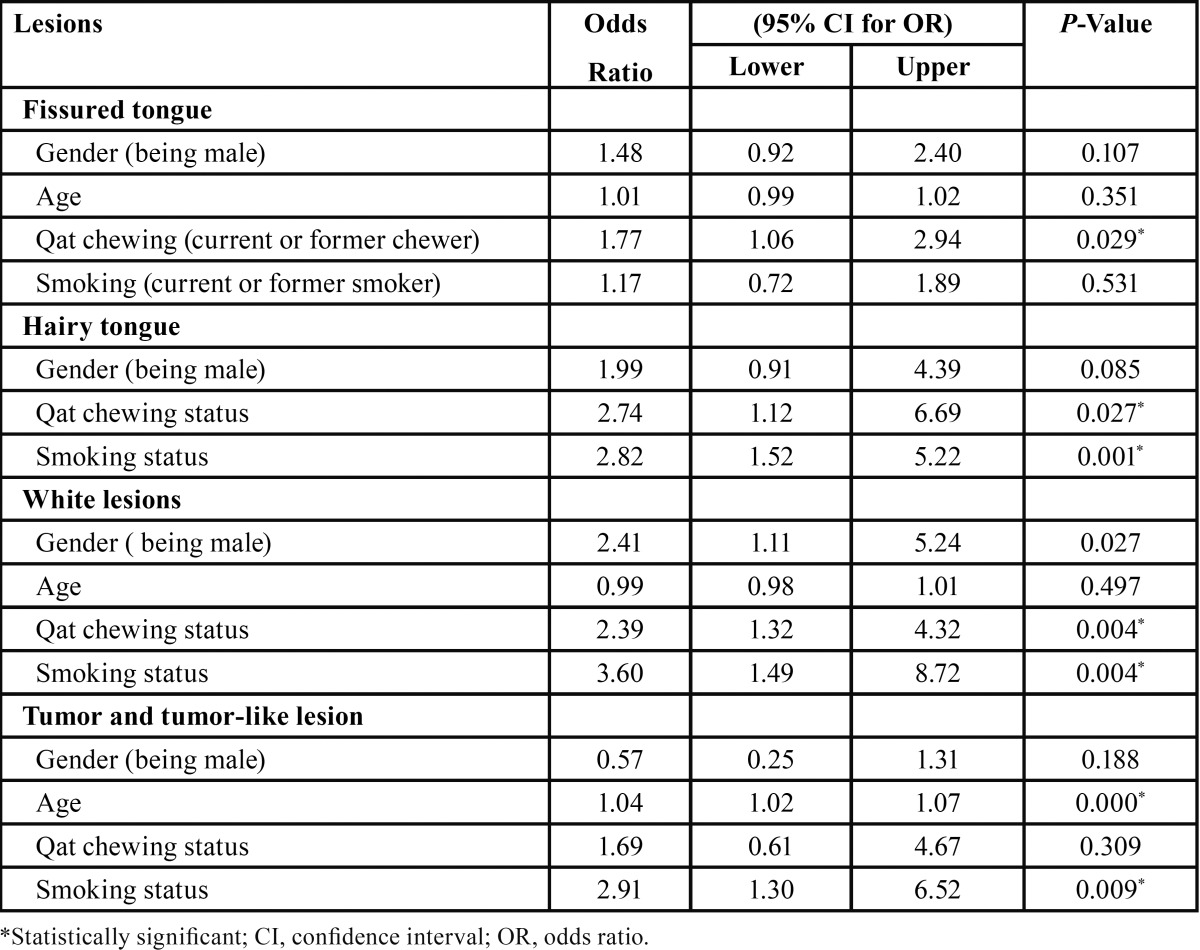
Association between oral lesions and variables according to the multiple logistic regression results.

## Discussion

Prevalence of a disease is usually determined by cross-sectional studies. To the best of our knowledge, this is the first study to provide data on the prevalence and risk factors of oral mucosal lesions (OMLs) among adults in Yemen.

In our study, the prevalence of OMLs was 58.4%, which is comparable to results from Kuwait (58.1%) (7), Slovenia (61.6%) (4) and Spain (58.8%) (13). However, this rate is much higher than that reported in Iran (19.4%) (6), Saudi Arabia (15.0%) (5) and Turkey (15.5%) (2). These variations may be from differences in the subjects studied, diagnostic criteria used and types of oral mucosal conditions included.

In accordance with most of previous studies, the present authors found OMLs more common among males than females (14-16). This is probably because of the fact that most of the reported lesions were tobacco and/or qat related lesions, and these habits are more commonly practiced by men. Also, similar to previous studies, our results showed a higher prevalence of OMLs in older patients, which emphasizes the importance of routine examination of oral mucosa, particularly in adults (5,7,15).

Epidemiological surveys have shown that tongue lesions constitute a considerable proportion of OMLs, and their prevalence varies in different parts of the world (2,13,17,18). Our study corroborates these findings, as fissured tongue and hairy tongue were among the most prevalent lesions that we identified.

The prevalence of fissured tongue (37.2%), was higher than that reported in Jordanian (11.5%) (19), Indian (14.9%) (Patil *et al.*, 2013) and Turkish (5.2%) populations (2), whereas it was lower than that reported among Libyan population (48.4%) (20). Fissured tongue has been suggested to be genetically determined and this could be the reason for such prevalence variability world-wide (21). Consistent with previous reports, the present authors found significant association between fissured tongue and increasing age. This can be explained by the fact that increasing age is associated with hyposalivation, which is one of the prime contributory factors (19).

Hairy tongue was seen in 15.9% of our study population. This percentage is higher than that described in Jordanian (2.4%) and Turkish (3.8%) populations (2,19). The present authors have also observed an association between occurrence of hairy tongue and qat chewing, which might explain the high prevalence of this lesion among Yemeni population. Further, similar to most previous studies, our study found it to be more frequent in males, elderly, and tobacco users (2,5,22).

Atrophic tongue papilla was reported in 2.9% of our study population. This figure is higher than the prevalence in Turkish population (0.7%) (2) and Malaysian dental patients (1.3%) (23). The prevalence in Indian dental patients was reported to be 11.5%, which is higher than the results of the present study (17). It is characterized by local or extensive loss of papilla and commonly seen in patients with nutritional deficiencies, xerostomia, trauma and candidiasis. The prevalence of geographic tongue in the present study was 1.5%, which is similar to those reported in Slovenian and South African populations (4,24). However, much higher prevalence was reported by many other studies (17,19).

White lesions were the second most common lesions seen in our study group. The most frequent white lesion was qat-induced white lesion (9.3%) followed by frictional keratosis (3.7%) and leukoedema (3.4%).

Qat-induced white lesion is a keratotic white lesion associated with the habit of qat chewing and strictly confined to the site of chewing (10,11,25). These lesions are confined primarily to the buccal mucosa and to less extent to vestibular and muccobuccal fold mucosa. Such lesions could be attributed to continuous friction of qat against mucosa during chewing session. However, the chemical components may play a role in inducing these lesions. Ali *et al.* (11) has suggested that these lesions intensify with increased duration and frequency of qat chewing. Our results support previous findings by other authors, who reported a high prevalence of white lesions among populations where qat chewing is a popular habit (10,11,25,26).

Although not significant, leukoedema was found to be more common among men than women. This finding (higher prevalence among men) supports previous findings by Jainkittivong *et al.* (22) and Reichart (27).

Traumatic ulcers were the most common ulcerative lesions (2.9%) followed by recurrent aphthous stomatitis, which is in accordance with other authors, who reported this lesion as the most common type of ulcerative lesions (2,5,7,22). The traumatic ulcers in this study population were most commonly located on the buccal mucosa and lateral surface of the tongue and mainly caused by trauma from fractured restorations and sharp edges of worn or carious teeth.

The present authors found fibroma to be the most common type of exophytic lesions, which is consistent with previous studies (2,5,7). The major cause of irritation fibroma is mechanical irritation from dentures, lip biting, calculus deposition, sharp margins of teeth, and long standing cheek biting and tongue thrusting. They can occur anywhere in the oral cavity, but as in our study, the tongue, buccal mucosa, and lip are the most frequent sites (7).

Physiological pigmentation was also a common finding in our study population. Several other studies have also reported it to be one of the most common lesions (7,13,28). We observed it mostly on the gingival tissue followed by the buccal mucosa, a finding similar to previous reports (7,28).

Denture stomatitis was not common in the present study, as we observed only one case (0.2%). This low rate contradicts findings from other studies in which denture stomatitis was reported to be one of the most common oral lesions (2,13,22). The percentage of denture wearers in the present study is very low ( Table 1) when compared to previous studies, which could be the reason for the low incidence of denture stomatitis and other denture related lesions in the present population.

Premalignant lesions were not common in our study population and the incidence was similar to previous reports. Leukoplakia was seen in 1% of our study population. This finding is similar to the prevalence reported in Brazilians (1.01%) (29), whereas this rate is much lower than that found in studies conducted in Slovenia (3.1%) (4) and Thailand (4.8%) (22). The prevalence of leukoplakia based on epidemiological data from different countries over the last 30 years varies from 1% to 13% with a mean value of 3% (30). Lichen planus was seen in 0.5% of our study population, a figure consistent with many previous studies (2,6). No oral cancer was observed in this study, which confirms the rarity of this lesion in the oral cavity. However, dental practitioners must remain alert for any suspicious lesions such as chronic non-healing ulcers, white patches with red areas and indurated lesions.

Additionally, multiple regression analysis results demonstrated a significant association between tobacco habits and keratotic white lesions, hairy tongue, and tumors and tumor-like lesions. These results confirm previous published reports (22,29,31-32). Further, the results showed a significant association between qat chewing and occurrence of certain oral lesions: white lesions (*P* < 0.01), fissured tongue (*P* < 0.05) and hairy tongue (*P* < 0.05). The association between mucosal white lesions and qat chewing has been extensively studied and confirmed by many previous studies (10,11,25). A recent study conducted by Schmidt-Westhausen *et al.* (26), has also shown a significant association between qat chewing and white lesions among Yemeni women. With respect to fissured and hairy tongue, the exact mechanism by which qat may affect the tongue is still unclear, as there is no data available in the literature. Yet, such lesions might be attributed to xerostomia induced by qat, which is one of the contributory factors for causing tongue conditions (19). Also, the chemical composition of qat and the pesticides used in the treatment of qat plants might play a role in causing and aggravating these lesions.

In conclusion, a large proportion of this population had one or more oral mucosal lesions. In agreement with previous studies, age, gender, and deleterious oral habits were associated with occurrence of OMLs. These results suggest that smoking and qat chewing cessation programs have the potential to improve oral health and decrease the incidence of oral cancer.
